# Academic Clinicians’ Workload Challenges and Burnout Analysis

**DOI:** 10.7759/cureus.6108

**Published:** 2019-11-08

**Authors:** Aussama K Nassar, Abdul Waheed, Faiz Tuma

**Affiliations:** 1 Trauma, Acute Care Surgery, and Critical Care, Stanford University School of Medicine, Stanford, USA; 2 General Surgery, Brandon Regional Hospital, Brandon, USA; 3 General Surgery, Central Michigan University College of Medicine, Saginaw, USA

**Keywords:** burnout, physicians, clinician, academic

## Abstract

Academic clinicians have high expectations to meet in their academic institutions. Accomplishments are to be expected in multiple domains for their positions’ sustainability and promotions. In addition to excelling in their clinical practice, they are expected to maintain productive scholarly activities and meet the required educational and administrative responsibilities. Striking a balance between clinical, educational, research, and administrative duties is highly challenging and could lead to emotional exhaustion and burnout. Lately, the ever-growing patient population, competitive academic environment, and resident work hour restrictions have led to increased strain and demand on academic physicians and predisposing them to burnout.

Despite the numerous studies looking at burnout in various professions, fewer studies have looked at burnout, specifically in clinical faculty members. Little is known about academic job satisfaction, stress, and rates of burnout, or how these factors affect scholarly success and productivity. Clinician faculty educators may be at significant risk of burnout. There is some evidence that clinically burned-out faculty had less confidence in their teaching skills and had fewer life-long learning habits. These results suggest that burnout may influence not only the quality of care but also the quality of training provided to others.

## Introduction and background

Burnout is a psychological-psychiatric term that was coined by H.J. Freudenberger, who defined burnout as ''the extinction of motivation or incentive, especially where one's devotion to a cause or relationship fails to produce the desired results”. Subsequently, Maslach and colleagues conceptualized burnout along with three subscales or dimensions: emotional exhaustion, depersonalization, and personal accomplishment [1]. Emotional exhaustion (EE) refers to feelings of being emotionally drained by one’s work. The most widely used instrument to measure burnout is Maslach and Jackson’s Burnout Inventory (MBI). The MBI scale is comprised of three subscales or dimensions: emotional exhaustion (EE), depersonalization (DP), and personal accomplishment (PA) [2]. EE occurs when an individual’s emotional resources are critically reduced. DP is characterized by negativism about clients. PA arises when workers feel unsatisfied with their job accompanied by a low sense of accomplishment and projecting that onto their clients [1]. Burnout syndrome is usually observed in jobs that involve human interaction [3]. Occupations such as healthcare and education have been extensively studied for burnout syndrome. The academic clinicians have given that they combine the two roles of being an educator and a physician, and that might increase the likelihood of burnout [4].

Academic clinicians comprise a unique subpopulation of clinicians. In addition to facing the high expectations of providing tertiary/quaternary care and clinical performance, they are also expected to carry out other non-clinical academic activities such as research, teaching, supervision of trainees, and additional administrative responsibilities [5]. Accomplishments in research and teaching are essential for academic promotions. This heavy workload must also be balanced delicately with clinicians’ personal lives [5].

Workload measurement systems to quantify clinicians’ workload were historically not well developed in clinical fields [6]. This is in contrast to allied healthcare professionals who have had systems in place for decades due to the salaried nature of their roles and accountability requirements [7]. In North America, physician-funding models for most of the faculty medical departments are production-based, fee-for-service, or relative value units (RVUs), equating more work to more income. The ability to act like a clinician, educator, researcher, and administrator means that they will need to allocate more time into direct patient care to reach the same income of non-academic physicians [8]. This production and fee-for-service systems may generate more workload, especially for junior faculty and staff members, adding more stress to their new roles.

The workload of academic clinicians falls into two main categories: clinical and non-clinical (i.e., teaching, research, and administration). It is difficult to compartmentalize the amount of work in each category. Still, a recent study reported that 70% to 75% of a physician’s time is spent on clinical activities, leaving 25% to 30% on non-clinical activities. Besides, junior physicians spent more time on total clinical activities than both the mid-career and senior physicians, although senior physicians spent more time on non-clinical activities than both junior and mid-career physicians [9].

Lately, the growing scarcity of resources combined with growing patient population and resident work hour restrictions has led to the increased patient-to-attending physician and patient-to-resident ratios. This change has been associated with higher perceptions of insufficient time for patient care, increased occupational stress, burnout, and more negative attitudes by clinicians regarding time for teaching and trainee satisfaction [10].

The multiple clinical and non-clinical roles of academic clinicians may lead to role conflict and role ambiguity. Role conflict exists when there are several roles for the same person, requiring different or incompatible behaviors. Role ambiguity arises when individuals are uncertain about their duties, authority, allocation of time, and relationships with others. Role conflict and role ambiguity can result in increased work-related stress, decreased productivity, and impaired organizational efficiency [10]. Both role conflict and uncertainty are significant causes of work-related stress and correlate to burnout [11]. Role conflict applies to academic clinicians as they must balance non-clinical activities on top of their regular clinical responsibilities. Role ambiguity in academic clinicians may be due to the fact they do not receive structured training on how to teach, which can lead to role confusion.

Despite the numerous studies looking at burnout in various professions, few studies have looked at burnout, specifically in clinical faculty members. A recent systematic review on the prevalence of burnout among physicians from 1991 to 2018 reported significant variability in the reported burnout prevalence ranging from 0-80.5%, with high variability on how burnout is defined and measured. Also, there was a lack of a strong association between burnout and demographic variables, specialties, and depressive symptoms [12]. Academic clinicians are a unique subset population of physicians; they are expected to perform additional roles compared to non-academic physicians. Their two leading roles are clinical (i.e., directly related to patient care) and non-clinical (i.e., indirectly related to patient care) such as education, research, and administration. On average, up to 24% of working hours were spent on administrative duties [12]. As mentioned earlier, these conditions would create work overload, role conflict, and role ambiguity and may be associated with additional chronic occupational stress and burnout.

Burnout is well studied in a variety of educators, including elementary, middle school, high school, and university teachers [10-14]. Similarly, a large amount of research has been directed towards the prevalence and effect of burnout within residents and staff physicians [13,14]. Combining the two roles of being an educator and a physician might increase the likelihood of burnout in academic physicians.

Burnout can start with the commencement of medical training or residency and can be carried over to the attending level. A study of United States medical students showed that 50% of medical students experienced burnout, and 10% experienced suicidal ideation, with a correlation being reported between burnout and suicidal ideation [14]. Another North American study reported high levels of emotional exhaustion and depersonalization in approximately 50% of internal medicine residents. International medical graduates (IMGs) showed lower levels of burnout compared to US medical graduates. A possible explanation was that IMGs have more resilient personality traits and have a lower tendency for burnout since they were able to successfully make it through the highly competitive selection process. Burnout is also correlated with higher levels of educational debts physicians are suffering from at the end of completing their residency [15].

## Review

Academic physicians may be at significant risk for burnout since many are practicing clinicians and researchers and are expected to handle the pressures associated with multiple roles simultaneously. Little is known about job satisfaction, stress, and rates of burnout in academic hospital medicine or how these factors affect scholarly success and productivity [15].

The relationship between clinician-educators’ duties and burnout was examined on a sample of internists, pediatricians, and family physicians practicing in an urban and underserved setting. They found that clinically burned-out faculty had less confidence in their teaching skills and had fewer life-long learning habits [16]. These results suggest that burnout may influence not only the quality of care but also the quality of training provided to others. Another study found that 23% of academic hospitalists had some degree of burnout, 67% reported high levels of stress, and 75% were satisfied with their jobs [17]. Predictors of low overall job satisfaction included training in a medical subspecialty, practicing at a non-university hospital, little satisfaction with the amount of personal/family time, amount of control over work schedule, and the level of support from their division chief. Predictors of burnout included low satisfaction with the amount of personal/family time and little satisfaction related to perceived control over work schedules [18].

There is a lack of studies that accurately delineate the sources of burnout to patient load, educational load, or administrative responsibilities. A study by Shanafelt et al. looked at career fit and burnout among 556 faculty physicians in the Department of Internal Medicine at an academic center [16]. This study demonstrated a 34% rate of burnout among faculty physicians, which was consistent with prior literature. The majority (68%) reported that patient care was most meaningful to them, with the fewest reporting administration (3%) as being useful. The time spent on the most significant activity was the largest predictor of burnout, whereby the more time faculty physicians focused on this aspect of work, the lower their risk of burnout. Also, burnout was associated with intent to leave academic medicine. However, this study focused only on a single profession (Internal Medicine) working at a single institution, which makes it difficult to generalize to other specialties and other institutions. Furthermore, other unmeasured factors (e.g., workload, autonomy, and administrative demands) may have contributed to burnout and the intent to leave academic medicine [16]. This would have an ever-lasting impact on patient care and the quality of education delivered at our academic centers.

## Conclusions

Academic clinicians are a unique subset of physicians that have higher expectations and responsibilities than other physicians and thus are at an increased risk of burnout syndrome. Burnout syndrome in academic clinicians is associated with psychological implications, disengagement, and reduced confidence. This has substantial repercussions on patient care as well as long-lasting impacts on the training for future physicians. There is a lack of studies exploring in-depth the direct relationship between academic physicians’ multiple roles and burnout. Studies in this area are required to solidify the contributing variables of burnout in our healthcare system.
